# Nitric oxide- induced *AtAO3* differentially regulates plant defense and drought tolerance in *Arabidopsis thaliana*

**DOI:** 10.1186/s12870-019-2210-3

**Published:** 2019-12-30

**Authors:** Murtaza Khan, Qari Muhammad Imran, Muhammad Shahid, Bong-Gyu Mun, Sang-Uk Lee, Muhammad Aaqil Khan, Adil Hussain, In-Jung Lee, Byung-Wook Yun

**Affiliations:** 1 Laboratory of Plant Functional Genomics Department of Plant Biosciences, Kyungpook National University, Daegu, Republic of Korea; 2 Laboratory of Plant Physiology, Department of Plant Biosciences, Kyungpook National University, Daegu, Republic of Korea; 3 Department of Agriculture, Abdul Wali Khan University, KPK, Mardan, Pakistan

**Keywords:** RNA-seq analysis, Nitrosative stress, ABA metabolism genes, Basal defense, *R*-gene-mediated resistance, Drought stress, Stomatal regulation

## Abstract

**Background:**

Exposure of plants to different environmental insults instigates significant changes in the cellular redox tone driven in part by promoting the production of reactive nitrogen species. The key player, nitric oxide (NO) is a small gaseous diatomic molecule, well-known for its signaling role during stress. In this study, we focused on abscisic acid (ABA) metabolism-related genes that showed differential expression in response to the NO donor *S*-nitroso-l-cysteine (CySNO) by conducting RNA-seq-based transcriptomic analysis.

**Results:**

CySNO-induced ABA-related genes were identified and further characterized. Gene ontology terms for biological processes showed most of the genes were associated with protein phosphorylation. Promoter analysis suggested that several *cis*-regulatory elements were activated under biotic and/or abiotic stress conditions. The ABA biosynthetic gene *AtAO3* was selected for validation using functional genomics. The loss of function mutant *atao3* was found to differentially regulate oxidative and nitrosative stress. Further investigations for determining the role of *AtAO3* in plant defense suggested a negative regulation of plant basal defense and *R*-gene-mediated resistance. The *atao3* plants showed resistance to virulent *Pseudomonas syringae* pv. *tomato* strain DC3000 (*Pst* DC3000) with gradual increase in *PR1* gene expression. Similarly, *atao3* plants showed increased hypersensitive response (HR) when challenged with *Pst* DC3000 (*avrB*). The *atgsnor1–3* and *atsid2* mutants showed a susceptible phenotype with reduced *PR1* transcript accumulation. Drought tolerance assay indicated that *atao3* and *atnced3* ABA-deficient mutants showed early wilting, followed by plant death. The study of stomatal structure showed that *atao3* and *atnced3* were unable to close stomata even at 7 days after drought stress. Further, they showed reduced ABA content and increased electrolyte leakage than the wild-type (WT) plants. The quantitative polymerase chain reaction analysis suggested that ABA biosynthesis genes were down-regulated, whereas expression of most of the drought-related genes were up-regulated in *atao3* than in WT.

**Conclusions:**

*AtAO3* negatively regulates pathogen-induced salicylic acid pathway, although it is required for drought tolerance, despite the fact that ABA production is not totally dependent on *AtAO3*, and that drought-related genes like *DREB2* and *ABI2* show response to drought irrespective of ABA content.

## Background

Nitric oxide (NO)—a small gaseous molecule is considered as the best signaling molecule in both plants and animals and deemed as the “Molecule of the Year” by *Science* in 1992 [1]. Numerous studies have been focusing on exploring its role in different life processes. Although the source and production of NO in animal cells are well-understood, its production particularly through oxidative pathways in higher plants is not yet known with certainty [2], and scientists have been attempting to identify a proper NO synthase in higher plants. NO is emerging as a key regulator of diverse plant processes such as growth, development, stomatal regulation, senescence, defense, and environmental interactions [2, 3].

Unlike classical signal transduction, NO and its chemical derivatives called reactive nitrogen species (RNS) act through chemical reactions with particular targets in different proteins [4]. *S*-nitrosocysteine (CySNO) and *S*-nitrosoglutathione (GSNO) are considered as two key NO donors as they spontaneously decompose in aqueous solutions releasing NO [5]. CySNO has the ability to infiltrate plant cells via specific l-type amino acid transporters [6], where it can undergo *S*-nitrosation, a type of post-translational modification in which the NO moiety is covalently attached to solvent-exposed cysteine residues forming *S*-nitrosothiols (SNOs). When NO is in excess, SNO allows the attachment of NO to glutathione to form GSNO, a relatively stable mobile reservoir of NO [7]. Thus, *S*-nitrosation is considered as the most relevant mechanism to transfer NO bioactivity that can consequently alter protein function [8]. Many proteins have already been reported to be *S*-nitrosated, although the list is continuously increasing [9]. These include Arabidopsis salicylic acid (SA)-binding protein 3 [10], non-expressor of pathogenesis-related gene-1 [11], and auxin receptor transport inhibitor response-1 [12]. Similarly, many proteins are regulated through *S*-nitrosation in response to abiotic stresses, such as AHb1 involved in hypoxia; Rubisco regulating low temperature stress; GAPDH in response to salt stress; APX, GR, and GAPDH in response to high light condition; CAT and GOX in response to cadmium stress (reviewed in [13]). Cellular SNO levels are regulated by a key enzyme GSNO reductase (GSNOR), mutation in which results in compromised growth and plant defense [14].

Drought stress is the main environmental factor that affects growth and productivity of plants worldwide. The damage due to water deficits is even greater than the combined damage of other environmental factors [15]. Drought condition results in evident phenotypic changes such as reduction in the growth of shoot and root and decrease in the diameter of the stem that disrupts plant-water relations with reduced water-use efficiency [16, 17]. The role of NO in the regulation of drought stress is also studied [18, 19]. Reports suggest that NO play key role in drought tolerance by enhancing the antioxidant systems, proline and osmolytes metabolism of several plant species [20, 21]. NO may also help indirectly in drought tolerance by restricting water loss via ABA-mediated stomatal response through various signaling cascades including mitogen-activated protein kinase (MAPK) etc. [22]. NO also play a multi-dimensional role in drought tolerance at molecular level by undergoing DNA methylation etc. Similarly, NO also regulates the expression of several genes responsive to drought stress including transcription factors (TFs) and antioxidant-related genes [23, 24]. Recently in an RNA-seq based transcriptomic study, [25] we reported several drought-related genes that showed differential expression in response to 1 mM CySNO, a NO donor. Similarly, several TFs that are induced by drought stress also showed response to NO. For example, NAC19 TF showed more than 150 fold change to CySNO [26]; this TF is reported to be induced by drought, high salinity and ABA [27]. Similarly, several members of MYB and WRKY TF families have also been reported to be differentially regulated in response to NO [26].

Abscisic acid (ABA), mostly referred to as a “stress hormone,” is the key phytohormone involved in the regulation of plant water status and stomatal movement. ABA biosynthesis involves two major genes zeaxanthin epoxidase [28] and 9-cis-epoxycrotenoid dioxygenase (NCED) [29, 30]. However, the last step that includes the oxidation of abscisic acid aldehyde by the gene family aldehyde oxidase (AO) plays a critical role in ABA synthesis. AOs consist of at least four genes (*AAO1, AAO2, AAO3, and AAO4*) [31]; among these genes, *AAO3* encodes AOδ, which has a high specificity for abscisic aldehyde [32]. Interactions between NO and ABA have also been studied; under drought tolerance, NO has been suggested to induce ABA production that further regulates stomatal responses [33]. This has been further confirmed using a reverse genetics approach wherein NO-deficient double mutant *nia1nia2* [KO mutation in nitrate reductase (NR)] was unable to close stomata in response to ABA, suggesting that NO is required for ABA-induced stomatal closure [34]. Another mechanism involving NO-mediated stomatal closure suggests that drought-induced ABA production activates NADPH oxidase, RBOHD, and RBOHF (respiratory burst oxidase homologs D and F) that produce superoxide burst, leading to the activation of NR for NO production that in turn activates mitogen-activated protein kinase (MAPK) signaling cascade to drive stomatal closure [2].

Previously, we reported thousands of genes that respond to the NO donor CySNO by conducting RNA-seq-based transcriptome analysis [25]. In this study, we focused on CySNO-induced ABA biosynthesis- and signaling-related genes. By using combined in silico and in vivo approaches, we showed the regulatory role of NO-induced ABA genes (specifically *AtAO3*) in plant defense and drought regulation.

## Results

### Identification and characterization of ABA-related genes

**Fig. 1:**
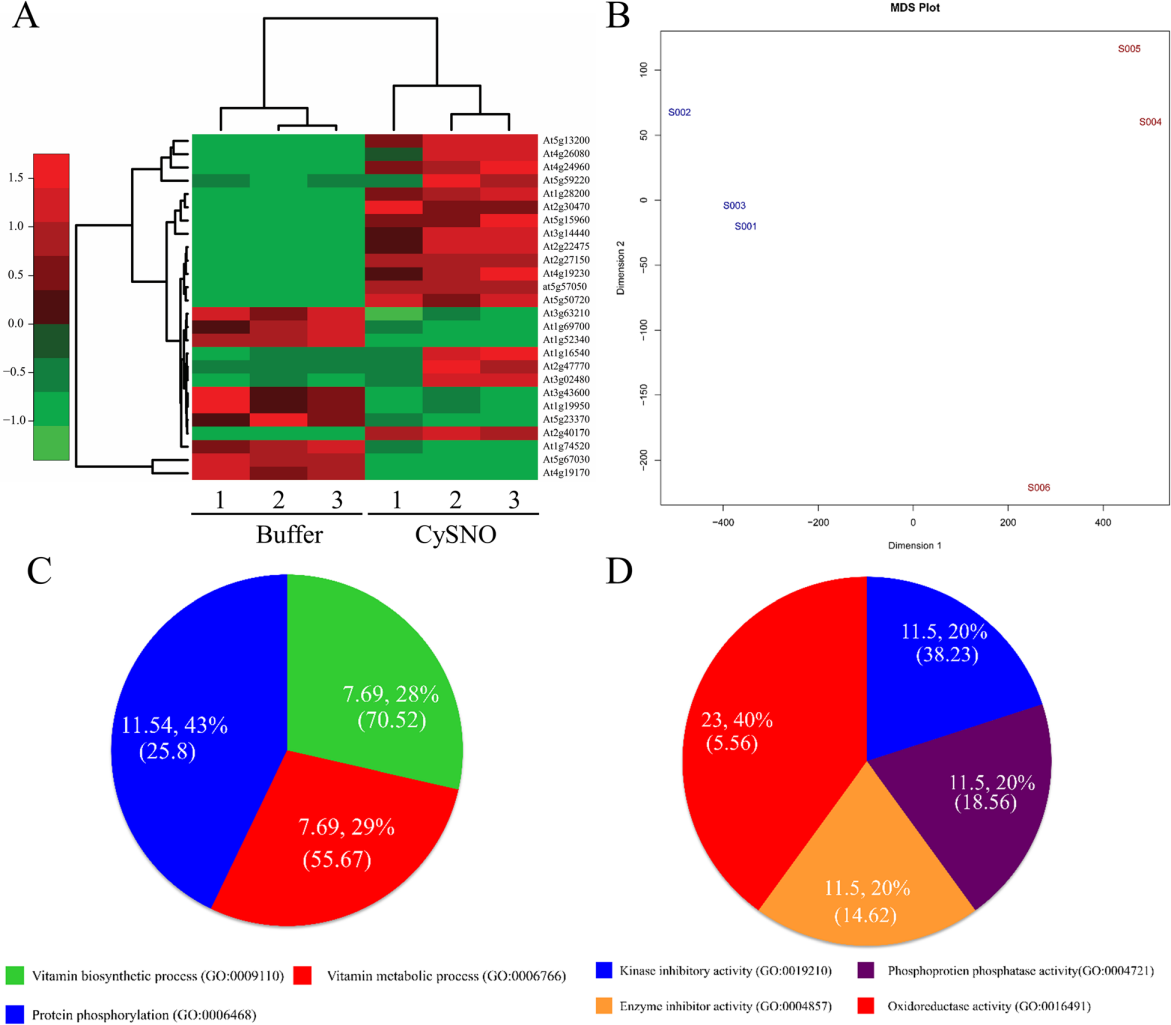
CySNO-induced ABA metabolism-related genes. Heatmap showing hierarchical clustering and expression values of differentially expressed ABA metabolism-related genes in RNA-seq-based transcriptome in response to 1 mM CySNO. (B) Multi-dimensional scatter (MDS) plot representing the dispersion in data. (C) GO terms for biological processes. (D) GO terms for molecular processes. The values in percentage represent percentage of genes found in the reference genome, whereas the ones in parentheses represent fold enrichment. The numbers in panel A represents three different replicates for buffer and CySNO treated samples. Similarly in panel B S001 to S003 are control samples whereas S004 to S006 are CySNO treated samples.

A total of 26 DEGs (17 up- and 9 down-regulated) involved in ABA metabolism that showed response to CySNO were found in the RNA-seq-based transcriptome. All the genes had at least 2-fold change in expression. The detailed list along with fold change and *p*-value is provided in Additional file 1. Among the up-regulated DEGs, the highest fold change was recorded for *Kin1* (At5g15960), which functions as an anti-freeze protein; according to TAIR description, transcript accumulation of this gene is induced by cold, ABA, and dehydration stress. Similarly, among the down-regulated DEGs, the highest fold change was recorded for *AtHVA22H* (At1g19950), which is an ABA-responsive gene. A Heatmap was generated to show the expression patterns along with a dendrogram to show the hierarchical clustering of CySNO-induced ABA metabolism-related genes (Fig. 1a). The MDS plot showing dispersion in data revealed that control samples had less dispersion, whereas the CySNO-treated samples showed slightly more dispersion (Fig. 1b). We further analyzed all the CySNO-induced ABA-related genes for GO terms of biological processes and molecular function to identify their fold enrichment and understand their putative functions. We found that, among the GO terms for biological processes, about 11.54% of genes against the total number of studied genes were annotated against protein phosphorylation with 25.8-fold enrichment (Fig. 1c). The highest fold enrichment (70.52 FE) among GO terms for biological processes was found for vitamin biosynthetic processes. Similarly, among the GO terms for molecular processes, the highest number of genes (23.40%) was involved in oxidoreductase activity, whereas the highest fold enrichment (38.23%) was found for kinase inhibitory activity (Fig. 1d).

### Promoter analysis for the presence of *cis*-regulatory elements

**Fig. 2:**
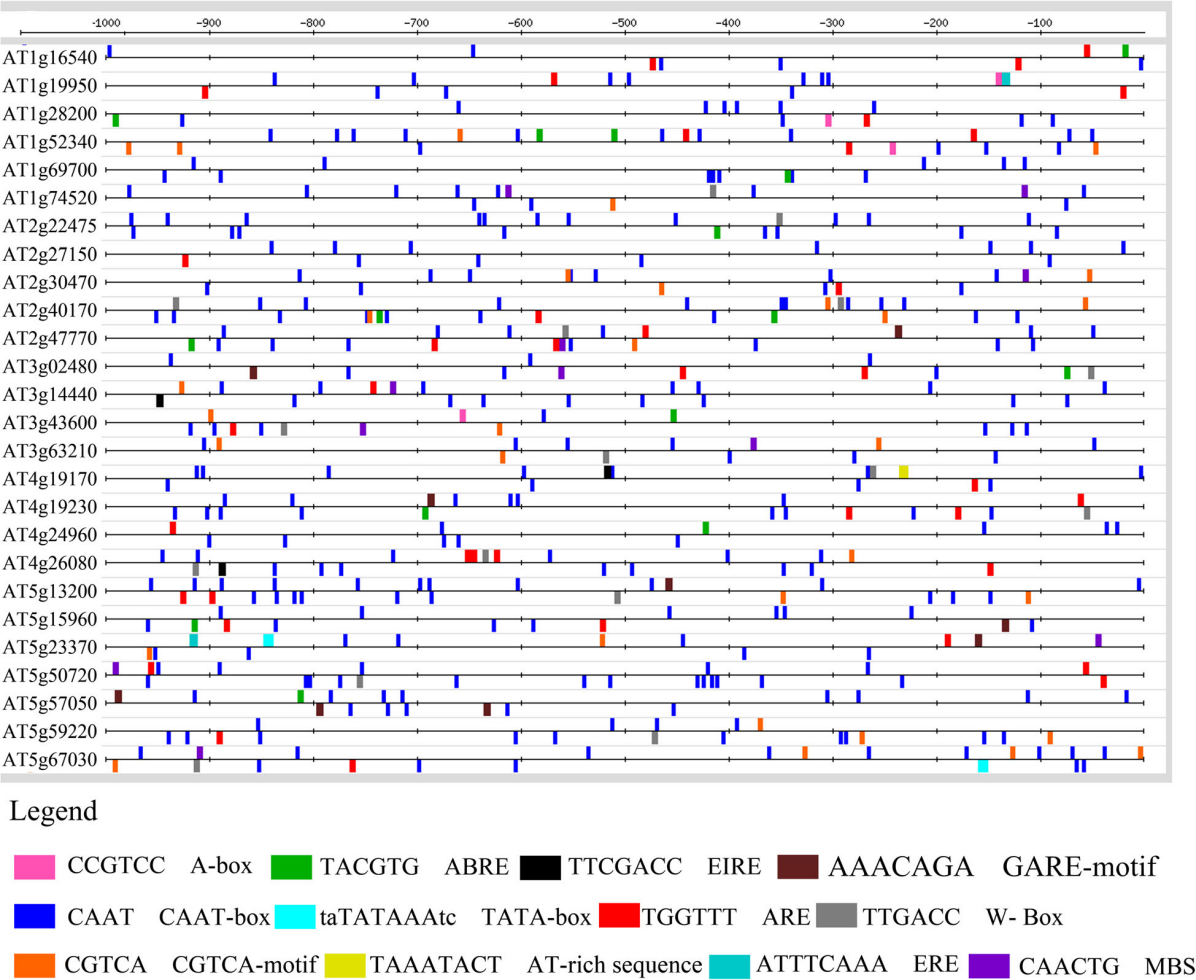
Promoter analysis of CySNO-induced ABA metabolism-related genes. Promoter sequences of NO-induced ABA-related genes 1 Kb upstream of the transcription initiation site were retrieved from TAIR (https://www.arabidopsis.org/) and analyzed using CARE to identify *cis*-regulatory elements present in the respective promoters. Selected *cis*-regulatory elements were then mapped using RSAT (http://rsat.eead.csic.es/plants/index.php).

ABA is mostly associated with abiotic stress conditions; however, significant crosstalk exists between biotic and abiotic stresses. Cao et al. [35] reported that ABA is not only important in mediating abiotic stress responses but also plays a pivotal role in plant defense responses. Therefore, we intended to analyze the promoter regions of CySNO-induced ABA-related genes. Many *cis*-regulatory elements were found in the promoters of different genes. For convenience, we selected 12 elements that were found in all genes and were involved in both biotic and abiotic stresses; we mapped them by using RSAT. These included CAAT-box that was found in 100% of the sequences; ABRE, found in 50% of the sequences; and GARE, found in 23% of sequences. The WRKY transcription factor-binding site (W-box) was found in 53.84% of sequences, which was the second most frequent *cis*-element found after CAAT-box. Another motif CGTCA was found in 50% of sequences, whereas MBS was found in 26.9% of the sequences (Fig. 2).

### AtAO3 differentially regulates root and shoot length under oxidative and nitrosative stress conditions

**Fig. 3:**
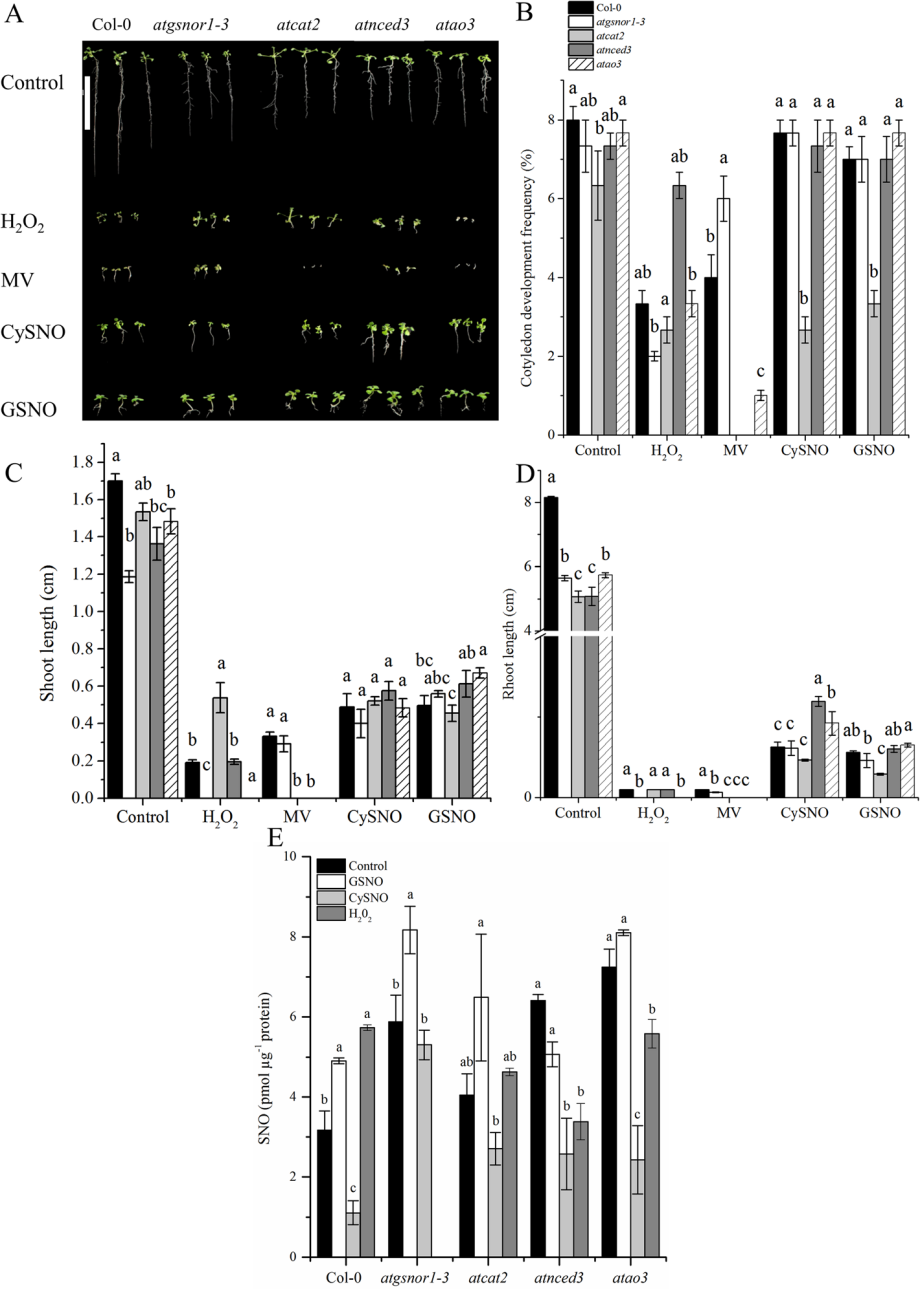
*AtAO3* showed differential regulation of shoot and root length under oxidative and nitrosative stress conditions. **a** Phenotypes of the indicated genotypes. **b** Cotyledon development frequency after one week under control, H_2_O_2_, and MV-mediated oxidative and CysNO- and GSNO-mediated nitrosative stress condition. **c** Shoot length and **d** root length of indicated genotypes under control, oxidative, and nitrosative stress conditions. All data points are the means of three replicates with each replicate pooled with 5 plants at least. The background in A was changed for more clarity. The Y-axis in D was interrupted for the better presentation of small values. The white scale bar in A is equal to 4 cm. Significant differences are represented by different letters suggesting differences between means at *p* ≤ 0.05 (DMRT). The experiment was repeated two times with similar results.

In this study, we focused on aldehyde oxidase (*AO*) that plays a critical role in ABA synthesis. Among the *AO* family genes, *AtAO2* and *AtAO3* showed differential response to NO. As mentioned in the introduction section AtAO3 encodes AOδ, which has a high specificity for abscisic aldehyde [32, 36]. Therefore, we used *AtAO3* knocked out (KO) mutant for downstream analysis. The possible role of *AtAO3* in plant growth and development was determined by analyzing growth parameters such as CDF and shoot and root length in WT and loss-of-function mutants *atao3* and *atnced3.* The *atgsnor1–3* deficient in AtGSNOR1 and *atcat2* deficient in AtCATALASE2 were used as control plants owing to their established role in plant growth and defense [14, 37]. The phenotypes of different genotypes under control and different stress conditions can be found in (Fig. 3a). Our results suggested no significant difference in CDF of *atao3* under control and nitrosative stress conditions compared to that in wild type (WT) (Fig. 3b). In contrast, under methyl viologen-mediated oxidative stress, *atao3* showed significant reduction in CDF (Fig. 3b). The *atgsnor1–3* mutant showed increased CDF compared to that in WT (Fig. 3b). Similarly, under nitrosative stress, *atao3* showed a donor-dependent response. In the case of CySNO-induced nitrosative stress, no significant difference was noted between *atao3* and WT; in contrast, the GSNO-mediated nitrosative stress resulted in significantly longer shoot length in *atao3* than that of WT (Fig. 3c). The root length of *atao3* was reduced under control and oxidative stress conditions, whereas increased under nitrosative stress (Fig. 3d). Thus, *AtAO3* positively regulates root length under control and oxidative stress, whereas negatively regulates it under nitrosative stress condition.

We further sought to determine total cellular SNO levels which is considered to comprise *S*-nitrosated proteins and low molecular weight SNOs including GSNO [38]. Although a reliable method for measuring GSNO levels is not developed yet but it is well-established that in biological systems, GSNO levels are congruent with total SNO levels [39]. For convenience, we selected plants exposed to only CySNO, GSNO and H_2_O_2_ as representative for nitrosative and oxidative stress respectively. Our results suggested that under control conditions *atnced3* and *atao3* showed significant increase (*p* ≤ 0.05) in cellular SNO levels compared to WT, whereas *atcat2* showed similar SNO levels to WT (Fig. 3e). After GSNO-mediated nitrosative stress, the cellular SNO levels in *atgsnor1–3* and *atao3* were significantly higher (*p* ≤ 0.05 and *p* ≤ 0.01 respectively) compared to WT (Fig. 3e). In mutant lines, *atcat2* and *atnced3,* the change in SNO levels was not significant. Similarly, CySNO treated plants showed reduced SNO levels in WT, whereas the SNO contents were increased in *atgsnor1*–*3*. The cellular SNO levels in *atcat2*, *atnced3* and *atao3* were almost similar under CySNO mediated nitrosative stress condition (Fig. 3e). In contrast, after H_2_O_2_-mediated oxidative stress, the cellular SNO levels in almost all genotypes were reduced compared to WT (Fig. 3e).

### AtAO3 negatively regulates plant basal defense

**Fig. 4:**
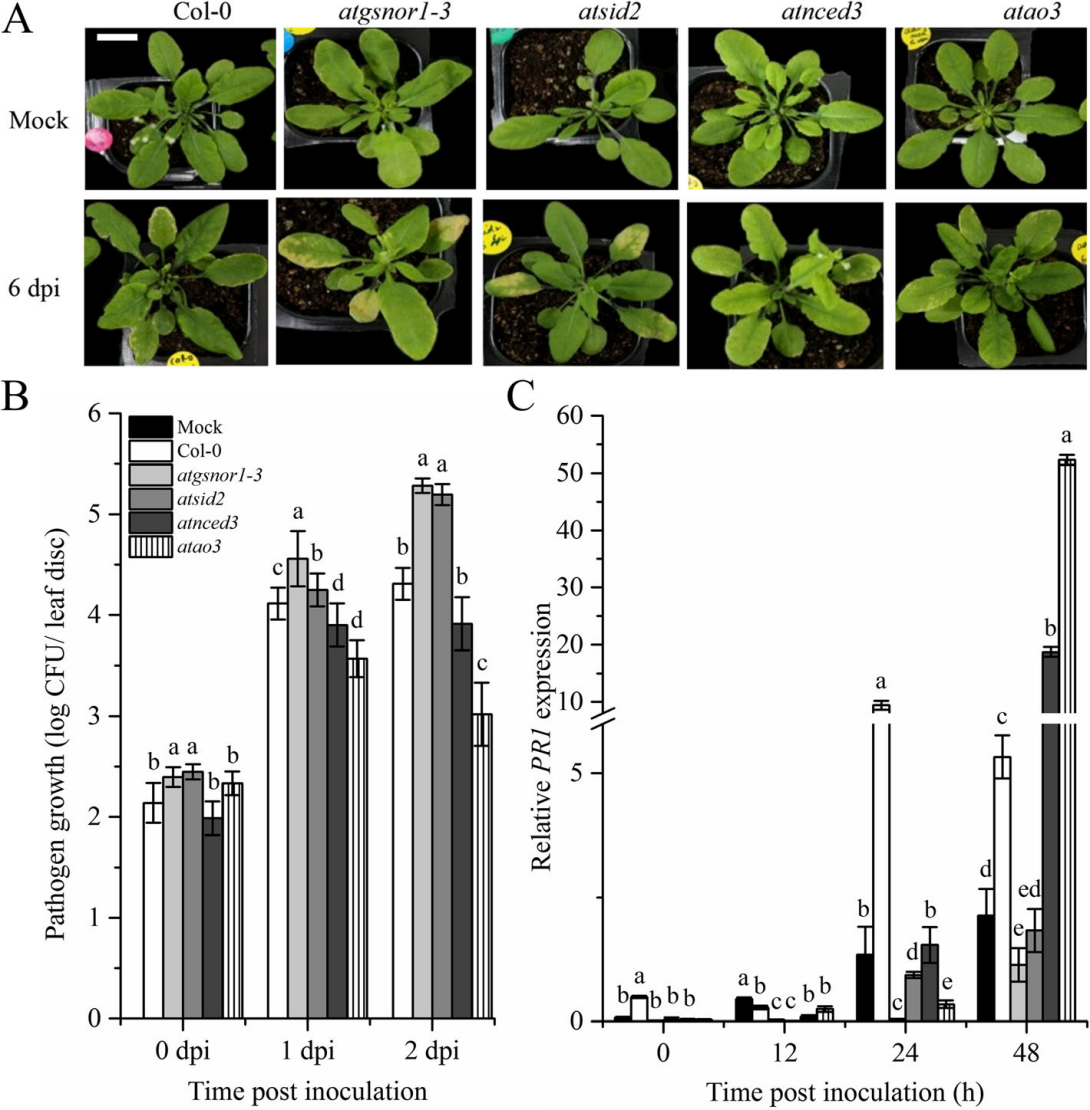
*AtAO3* negatively regulates basal defense. **a** Symptom development at 6 dpi. **b** Pathogen growth from infiltrated leaves. **c** Relative *PR1* expression after attempted virulent *Pst* DC3000 at 5 × 10^5^ colony forming units (CFU) mL^− 1^. The data points in panel (**b**) are the mean of five replicates, whereas those in panel (**c**) are the mean of three replicates. Background in panel (**a**) was modified for more clarity and white bar represents scale bar equal to 1 cm. The Y-axis of panel (**c**) was interrupted to clarify the small values. The experiments were repeated at least three times. Error bars represent ±SE. Different letters represents significant differences between means at *p* ≤ 0.05 (DMRT).

Whether *AtAO3* is involved in basal defense was determined by inoculating WT, *atao3, atnced3, atgsnor1–3,* and *atsid2* plants with virulent *Pst* DC3000 to assess the phenotypic response of all the genotypes toward virulent pathogens. We found that, at 6 days post-inoculation (dpi), *atao3* showed resistant phenotype, whereas *atgsnor1*–*3* and *atsid2* showed susceptible phenotype (Fig. 4a). We further investigated pathogen growth in WT and *atao3* along with relevant control mutants. Our results suggested that, at 0 dpi, no significant difference in bacterial growth was noted in all genotypes studied; however, at 1 and 2 dpi, *atao3* showed a significant reduction in pathogen growth compared to that in WT (Fig. 4b). Further, *atgsnor1*–*3* and *atsid2* showed increased pathogen growth at 1 and 2 dpi. However, no significant difference in pathogen growth was noted between *atnced3* and WT (Fig. 4b). The response of plants during biotrophic pathogen attack is mostly mediated by the key plant hormone SA [40]. Therefore, we aimed to determine the role of *AtAO3* in SA pathway signaling by determining transcript accumulation of *PR1* and *PR2*, the key marker genes related to the SA pathway. At early time points, *atao3* showed reduced transcript accumulation of *PR1*; however, at 48 h of inoculation, a robust increase in transcript accumulation of *PR1* gene compared to that in WT was noted (Fig. 4c). Moreover, *atgsnor1–3* and *atsid2* showed reduced transcript accumulation compared to that in WT at all-time points. All the genotypes showed reduced *PR2* expression over time compared to that in WT (Additional file 2).

### AtAO3 negatively regulates R-gene-mediated resistance

**Fig. 5:**
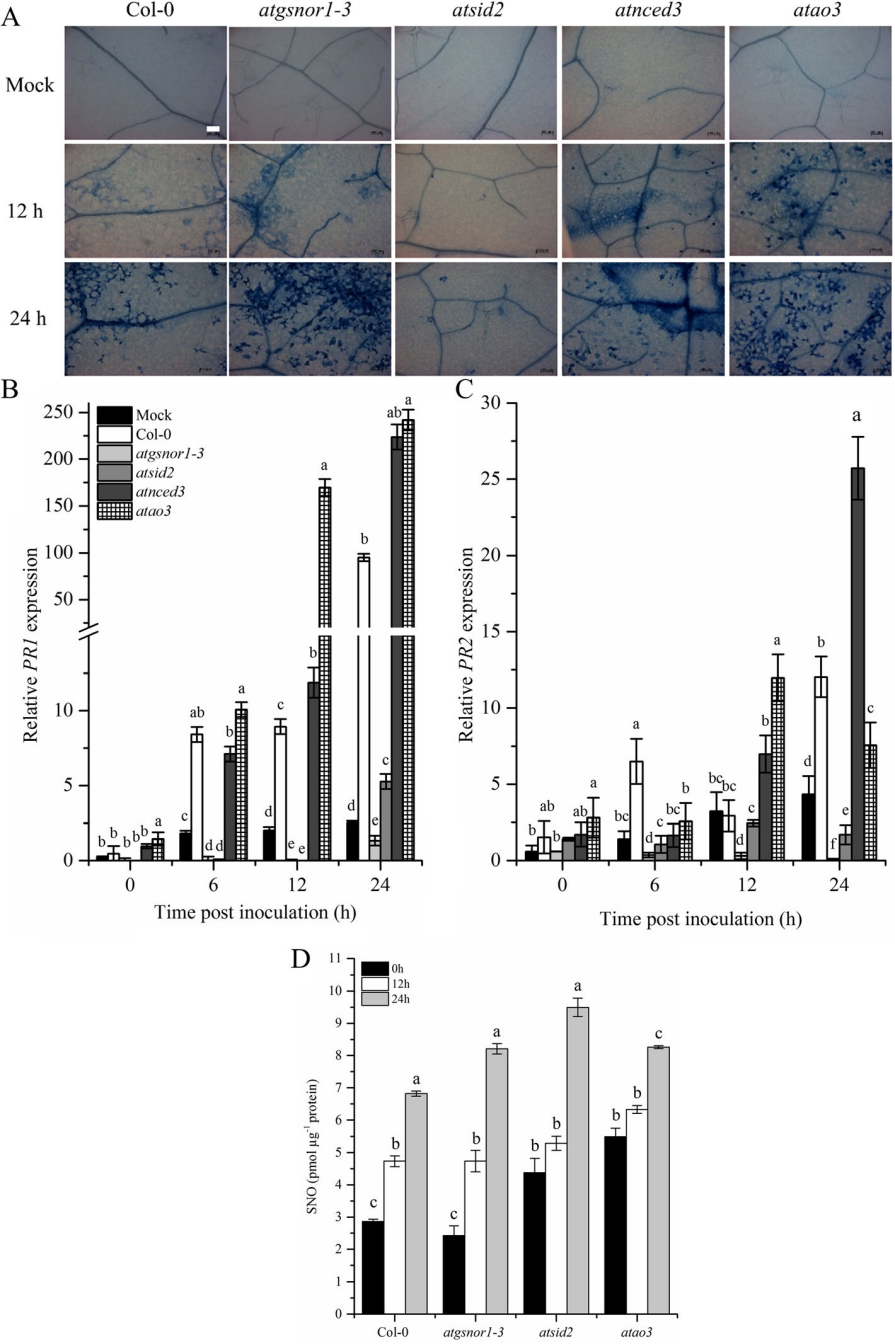
*AtAO3* negatively regulates *R-gene-*mediated resistance. **a** *ataao3* showed induced HR after *Pst* DC3000 (*avrB*) inoculation. **b** and **c** Relative transcript accumulation of *PR1* and *PR2* genes analyzed using quantitative real time PCR (qPCR) and (**d**) SNO measurement after *Pst* DC3000 (*avr*B) inoculation. The white small bar in WT mock plant in panel (**a**) represents scale bar and is equal ot 100 μM. The Y-axis of panel (**b**) was interrupted to clarify the small values. The expression was normalized against constitutively expressed *Actin*. Error bars represent ±SE. Different letters represents significant differences between means at *p* ≤ 0.05 (DMRT).

Plants use *R* genes encoded by the nucleotide-binding site-leucine-rich repeats (NBS-LRRs) that recognize pathogen-released effector molecules, thereby inducing *R*-gene-mediated resistance [41]. The ultimate fate of this resistance is HR, at the site of infection that restricts pathogens within the infected tissues. We inoculated WT, *atao3* mutants, and relevant controls with *Pst* DC3000 (*avrB*), and HR was observed using trypan blue staining. Our results suggested increased HR in *atao3*, followed by that in *atnced3* and *atgsnor1–3*, whereas *atsid2* showed almost no HR after 12 h post-inoculation (Fig. 5a). However, increased HR was observed for *atgsnor1–3*, followed by *atao3* and *atnced3* after 24 h of inoculation (Fig. 5a). We further investigated transcript accumulation in infected leaves following pathogen inoculation and found that *atao3* showed increased *PR1* expression at 6, 12, and 24 h after inoculation (Fig. 5b). *PR1* transcript accumulation was reduced for *atsid2* and *atgsnor1*–3 (Fig. 5b). In *atao3*, *PR2* transcript accumulation increased only at 12 h post-inoculation (Fig. 5c).

We also studied the cellular SNO levels in *atao3* and mutant lines used as comparative controls such as *atgsnor1–3* and *atsid2* after *Pst* DC3000 (*avrB*) inoculation. Our results suggested that the cellular SNO levels of *atao3* were significantly higher overtime compared to WT (Fig. 5d). Similarly, *atgsnor1–3* were slightly reduced at 0 h remains same to WT at 12 h while significantly increased at 24 h of pathogen inoculation. In case of *atsid2* the cellular SNO levels were increased at 0 and 24 h of inoculation while no significant difference was observed at 12 h post inoculation (Fig. 5d).

### atao3 shows differential response to systemic acquired resistance

**Fig. 6:**
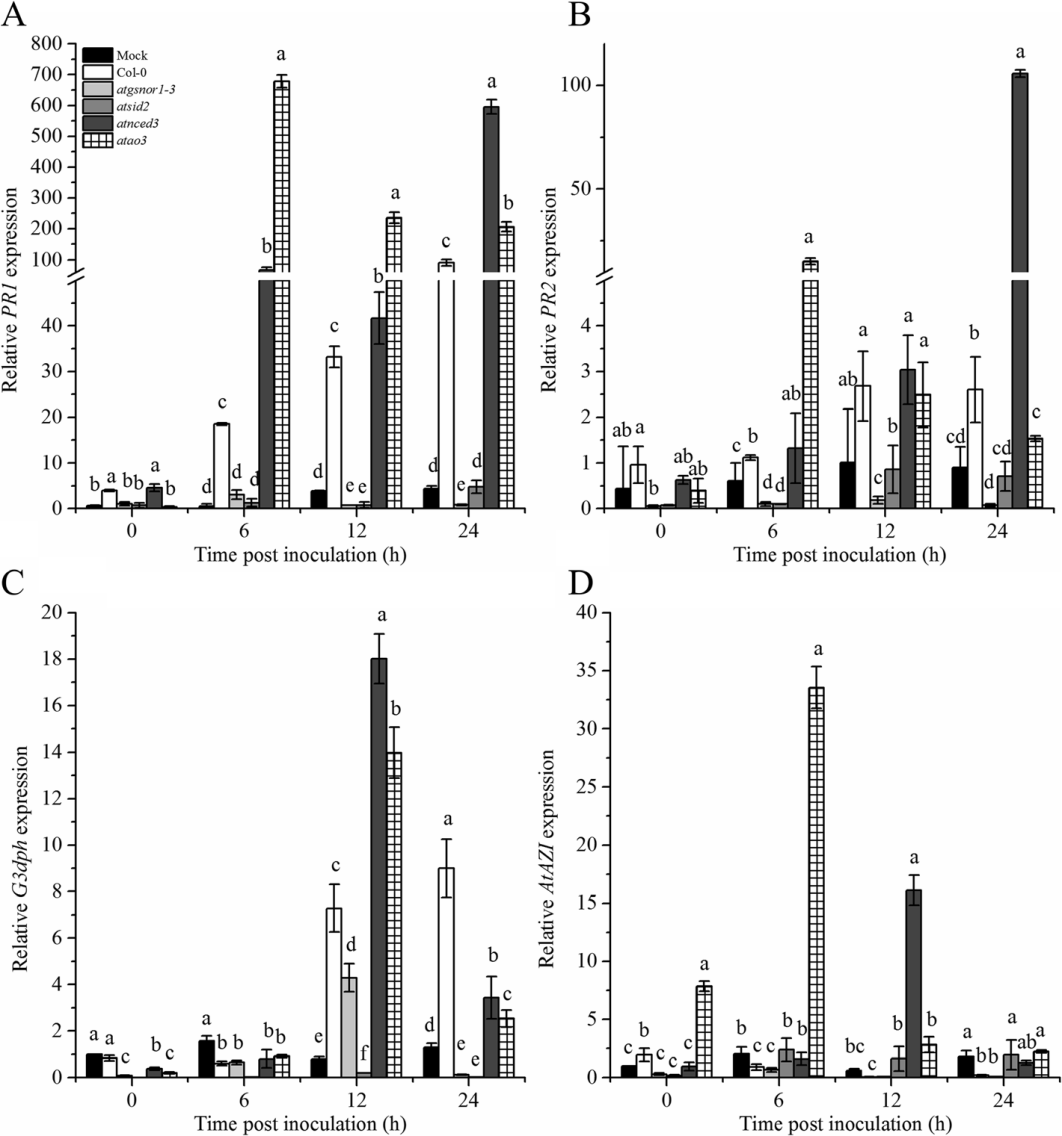
*AtAO3* negatively regulates systemic acquired resistance (SAR). Relative transcript accumulation of (**a**) *PR1* gene, (**b**) *PR2* gene, (**c**) *G3DPH*, and (**d**) *AZI* genes analyzed using quantitative real-time PCR (qPCR) in systemic leaves after *Pst* DC3000 (*avr*B) inoculation. The Y-axis of panels (**a**) and (**b**) was interrupted to clarify the small values. The expression was normalized against constitutively expressed *Actin*. Error bars represent ±SE (*n* = 3). Significant differences are represented by different letters (*p* ≤ 0.05) calculated by DMRT.

Systemic acquired resistance (SAR), an important defense system, is induced by signals initiated at the site of infection to other plant parts. NO, and ROS are reportedly involved in SAR activation [42, 43]. Therefore, we investigated whether AtAO3 played a role in SAR and inoculated plants with *Pst* DC3000 (*avrB*) at 5 × 10^6^ CFU. Leaf samples were collected from systemic leaves after pathogen inoculation, and transcript accumulation of key SAR marker genes such as *PR1*, glyceraldehyde 3-phosphate dehydrogenase (*G3PDH*), and azelaic acid inducer (*AZI*) was analyzed over time. The *PR1* gene transcript accumulation was significantly higher after 6 h and 12 h in the systemic leaves of *atao3*, followed by that in *atnced3*, compared to WT (Fig. 6a). In contrast, mock-treated plants showed reduced *PR1* transcript accumulation in all genotypes studied (Fig. 6a). Similarly, *atgsnor1–3* and *atsid2* barely showed *PR1* expression (Fig. 6a). *PR2* expression was high at 6 h in *atao3* leaves, whereas no significant difference was found at other time points (Fig. 6b). Furthermore, *G3PDH* showed increased transcript accumulation at 12 h after inoculation in *atao3* systemic leaves (Fig. 6c). The susceptible mutant lines *atgsnor1*–*3* and *atsid2* showed decreased expression compared to that in WT, whereas *atnced3* showed maximum transcript accumulation at 12 h after inoculation (Fig. 6c). Similarly, another SAR marker gene *AtAZI* was differentially expressed over time. At early time points, i.e., 0, 6, and 12 h after inoculation, *atao3* showed increased *AZI* expression compared to that in WT, whereas its expression was reduced at 24 h (Fig. 6d). Similar to *G3DPH*, *AZI* showed increased transcript accumulation in *atnced3* at 12 h (Fig. 6d). Both *atgsnor1*–*3* and *atsid2* showed decreased *AZI* expression over time (Fig. 6d).

### AtAO3 is required for drought tolerance through possible ABA-mediated stomatal regulation

**Fig. 7:**
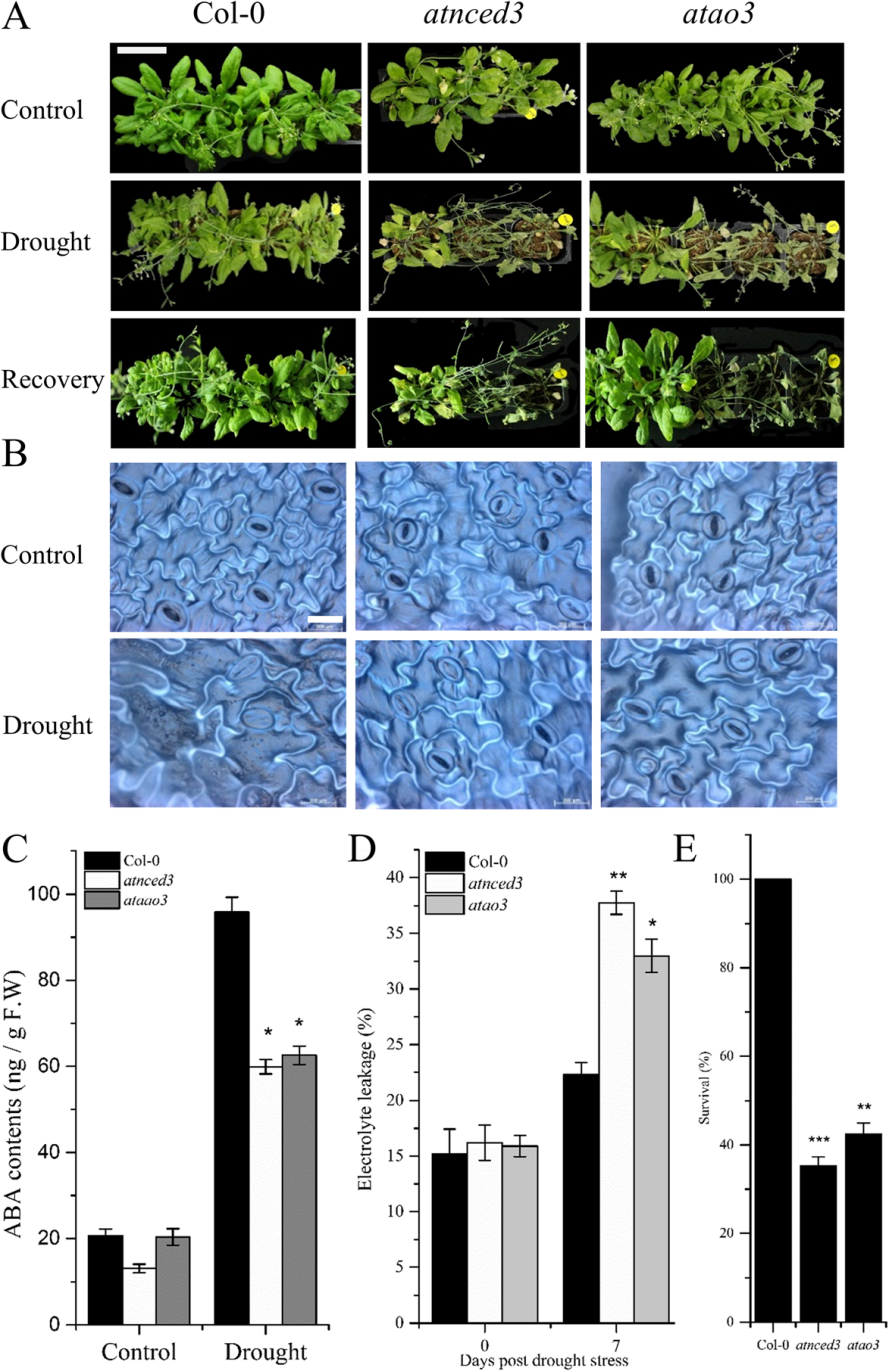
*AtAO3* is required for drought tolerance. **a** Phenotypes of the indicated genotypes under control, drought, and recovery stage. **b** Stomatal structure in the indicated genotypes under control and drought stress. **c** ABA contents (from fresh samples) in response to drought stress and control conditions. **d** Electrolyte leakage (%) under control and drought conditions (**e**) Survival percentage after re-watering the plants for 24 h. The white bar in panel A represents scale bar and is equal to 4 cm while in B is equal ot 200 μM. Error bars represents ±SE. Significant differences are represented by asterisks (Student *t*-test). * represents *p* < 0.05, ** represents *p* < 0.01 and *** represents *p* ≤ 0.001.

AtAO3 is involved in the very last step of ABA biosynthesis. ABA-induced NO production is known to regulate stomatal movements during drought stress [33, 44]. Therefore, we intended to investigate the possible role of NO-induced *AtAO3* in drought stress regulation. About 4-week-old WT, *atnced3*, and *atao3* mutants were subjected to drought stress by withholding water for 7 to 10 days. Both *atao3* and *atnced3* showed severe drought symptoms compared to WT (Fig. 7a). Mild drought symptoms such as wilting of leaves were noted earlier (at 3 days of stress) in *atao3* and *atnced3* mutant plants than in WT.

Stomatal regulation plays a key role in limiting water loss during drought stress. We hypothesized that the rapid drought symptoms such as wilting might be because of the inability of *atao3* to close stomata. Therefore, we visualized the stomatal structure in WT and *atao3* lines under control and drought-stressed plants. WT plants showed closed stomata, whereas the stomata of both *atnced3* and *atao3* remained open (Fig. 7b). Furthermore, the stomatal opening was quantified using ImageJ program. Our results suggested that, at 7 days post drought (dpd), the *atao3* mutant showed significantly (*p* ≤ 0.0001) higher number of opened stomata compared to WT (Additional file 3).

Similarly, drought-induced ABA production is critical for stomatal regulation [45]. *AtAO3* is involved in the last step of ABA biosynthesis; thus, theoretically, the loss of function of this gene should produce less ABA compared to that in WT. Therefore, we determined ABA levels in WT and knockout lines *atao3* and *atnced3* under control and drought stress. Our results suggested that, under control conditions, *atnced3* showed reduced ABA contents compared to that in WT, whereas no significant difference was found between *atao3* and WT. In contrast, at 7 dpd, significant (*p* ≤ 0.05) reduction in ABA contents was noted in *atao3* and *atnced3* compared to that in WT (Fig. 7c). We further investigated the cell membrane stability or electrolyte leakage and found that, at 0 dpd, no significant difference was noted in electrolyte leakage among all genotypes, including WT. However, at 7 dpd, significant increase in electrolyte leakage was observed in *atao3* and *atnced3* mutants (Fig. 7d). We further determined the recovery rate of different genotypes; we re-watered the plants and observed them after 24 h. We found that the survival rate of *atao3* and *atnced3* was less than that of WT (Fig. 7e). All these results suggests that AtAO3 is required for ABA production specifically under drought condition. The opened stomata in *atao3* KO line after drought stress suggested that their closing ability was impaired partially because of low ABA production in *atao3* and *atnced3* under drought conditions.

### Transcript accumulation of ABA biosynthesis and drought signaling genes

**Fig. 8:**
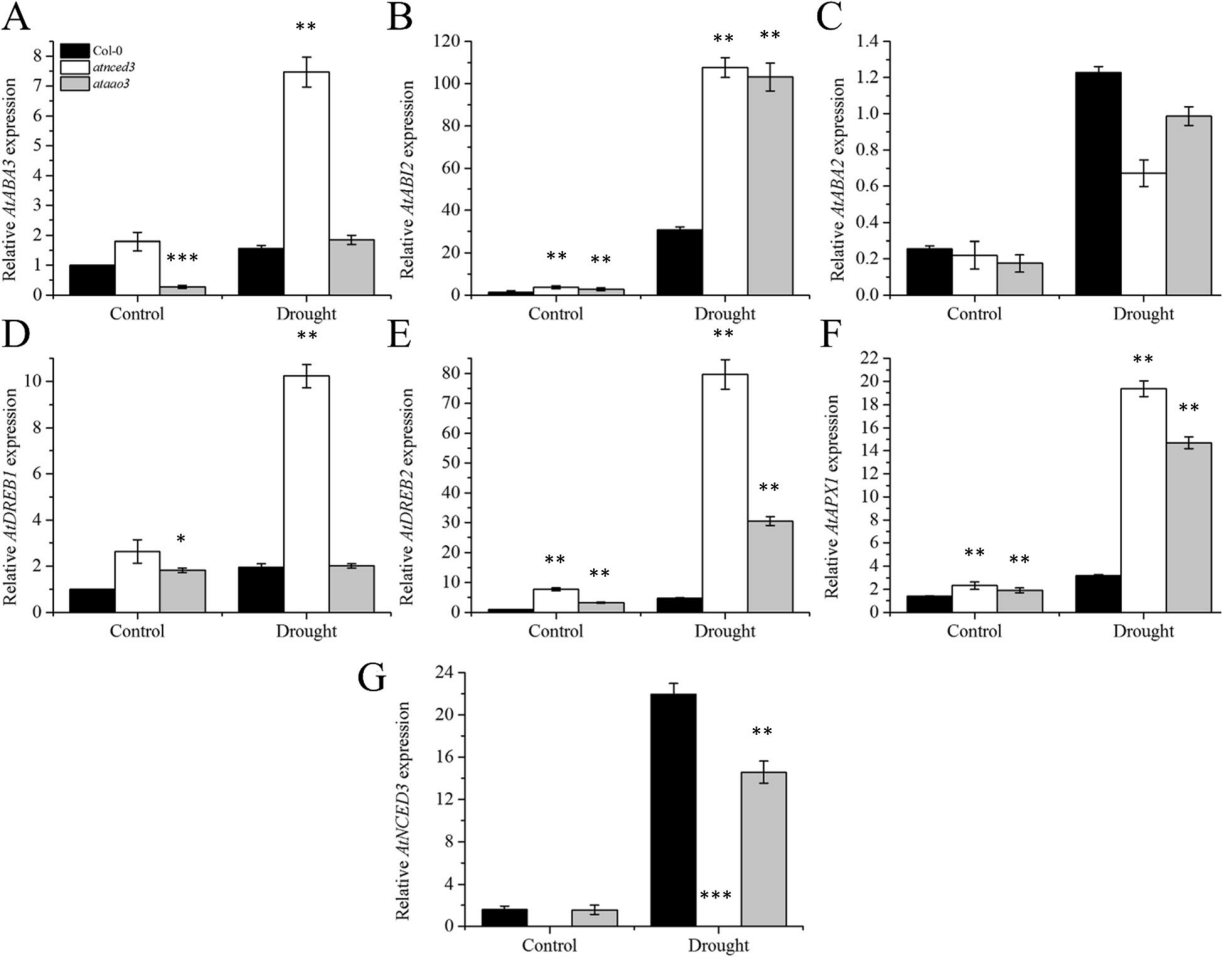
Expression of ABA biosynthesis and drought signaling-related genes in the indicated genotypes. Relative expression of **a** *AtABA3*, **b** *AtABI2*, **c** *AtABA2*, **d** *AtDREB1*, **e** *AtDREB2*, **f** *AtAPX1*, and **g** *AtNCED3.* The experiments were repeated at least three times. Error bars represent ±SE. Significance differences are represented by asterisks (Student *t*-test). * represents *p* < 0.05, ** represents *p* < 0.01 and *** represents *p* ≤ 0.001.

Next, we determined whether the loss of function of *AtAO3* affects other ABA biosynthetic genes. We selected the key biosynthetic genes such as *AtABA2*, *AtABA3*, and *AtNCED3* and analyzed them by using real-time qPCR. Our results suggested that except *ABA3*, which showed no significant difference compared to that in WT, the remaining ABA biosynthesis-related genes showed reduced transcript accumulation in *atao3* plants compared to WT (Fig. 8 A, C, and G). However, genes that were involved in ABA signaling or drought regulation such as *AtABI2, AtDREB2*, and *AtAPX1* showed increased transcript accumulation in *atao3* and *atnced3* compared to that in WT (Fig. 8 B, D, E, and F). *AtDREB1*showed no significant difference in transcript accumulation after drought stress compared to WT (Fig. 8d).

### Discussion

Both ROS and RNS are redox-active molecules that are produced after environmental insults. These molecules have the ability to inactivate cellular antioxidant system, thereby changing the redox status of cells [46]. The latter is less explored and has gained attention of scientists in the last couple of decades because of their remarkable regulatory role in various plant processes. Produced from NO, a small redox-active molecule having high diffusivity, RNS regulates many cellular processes such as stomatal regulation, seed germination, disease resistance, and plant responses to abiotic stresses [33, 47–49]. NO has the ability to control physiological processes directly by regulating the transcriptional machinery of certain genes. The global changes in gene expression in response to NO has been investigated using microarrays [50], RNA-seq [25, 51], and qRT-PCR [52].

**Fig. 9:**
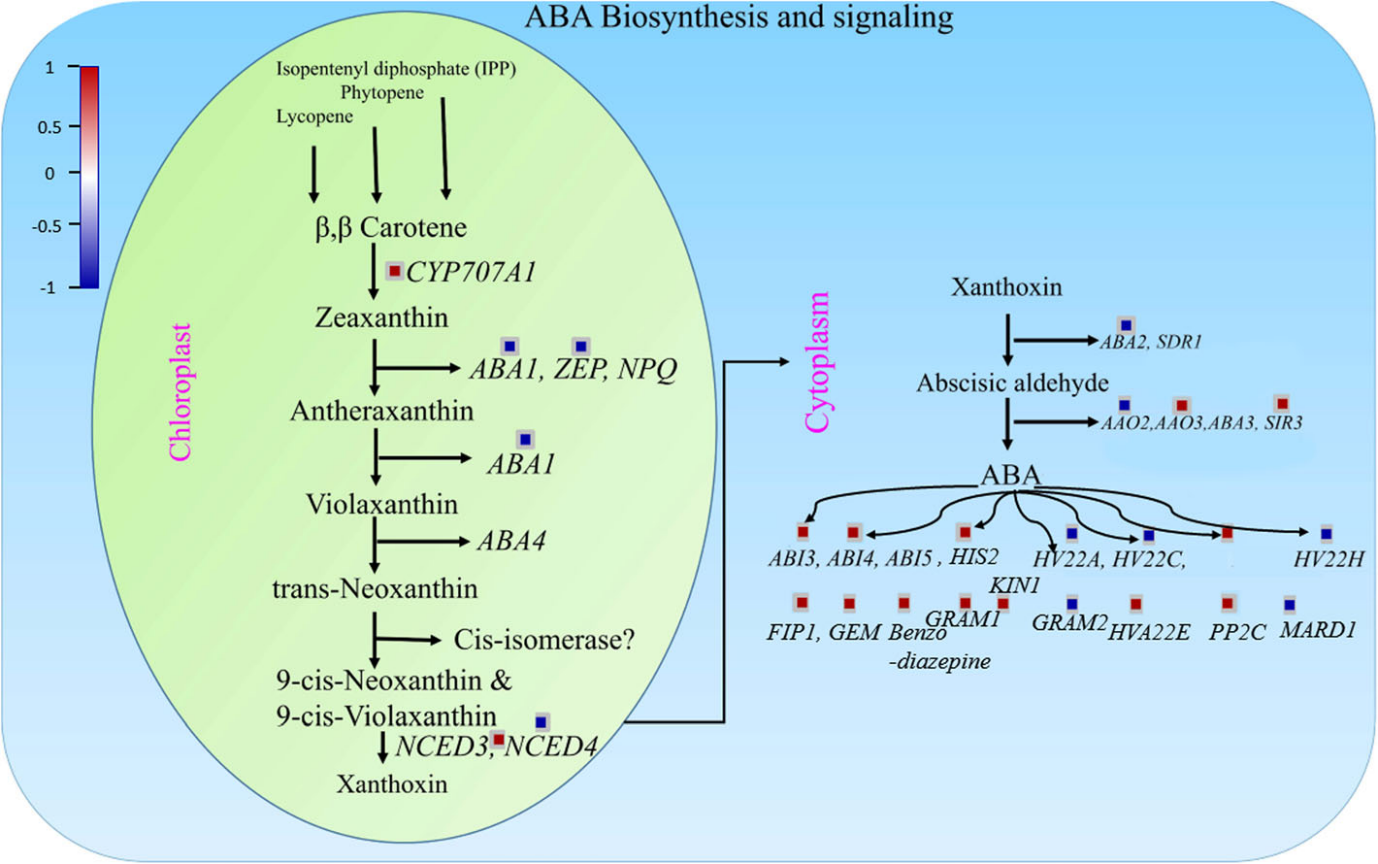
MapMan analysis showing up- and down-regulated ABA-related genes. Schematic presentation of ABA biosynthesis and signaling during drought conditions. The color key represents a scale of fold change from − 0.5 to 1. Red color represents up-regulated, whereas blue color represents down-regulated ABA-related genes in response to CySNO.

The Arabidopsis AO gene family consists of 4 alleles (*AAO1–4*); they are considered vital in ABA biosynthesis as they catalyze the last step in ABA biosynthesis involving the oxidation of abscisic aldehyde [36]. In this study, we focused on ABA-metabolism-related genes in *A. thaliana* that showed differential response to CySNO, a common NO donor by using RNA-seq-based transcriptomic analysis. The MDS plot was generated to detect dispersion in data, and we found that one replicate in treated samples showed dispersion, which might be because of low basal levels of gene expression in that replicate (Fig. 1b). All the genes were involved in either ABA biosynthesis or signaling (Fig. 9). Interestingly, most of the ABA biosynthesis-related genes were down-regulated, whereas most of the signaling genes were up-regulated in response ot NO donor (Fig. 9). Previous studies also showed differential expression of ABA-related genes. A study involving ozone treatment [53] reported about 14 genes involved in ABA metabolism that were also found in our transcriptome. This indicates that significant crosstalk exists between genes responding to ROS and NO, as suggested by [54]. The GO terms for biological processes revealed most of the genes (11.5443%) associated with protein phosphorylation (Fig. 1c), which is one of the important post-translational modification that regulates protein function in response to internal and external stimuli [55]. This further supports the notion that NO can potentially cause substantial post-translational changes in some proteins. NO is believed to transfer its bioactivity in biological systems through *S*- nitrosation [38]. Similarly, in GO terms for molecular function, the majority of the genes (23.40%) were associated with oxido-reductase activity (Fig. 1d). Oxido-reductases are key enzymes that regulate NO production and/or signaling [48, 56].

Many *cis*-regulatory elements were found in the promoter regions of CySNO-induced ABA metabolism-related genes at variable distances from the transcriptional start point. These include ABRE (TACGTG) involved in the regulation of drought response genes [57], EIRE (TTCGACC) involved in maximal elicitor-mediated activation of genes [58], and ERE (ATTTCAAA, ethylene-responsive elements regulating ethylene-mediated responses as the name indicates) [59]. Some of the important drought response genes are MBS (CAACTG MYB-binding site), which is involved in drought stress regulation [60], and W-box (TTGACC), the binding site for WRKY transcription factors [61] (Fig. 2), indicating that CySNO-induced ABA-related genes might be involved in the mechanistic control of genes associated with biotic and abiotic stresses. Bioinformatics approaches have revealed that NO-responsive genes contain a significantly higher number of transcription-binding sites in their promoters [24].

The *atao3* mutant showed differential response to oxidative and nitrosative stress in terms of shoot and root growth (Fig. 3a, c, d). The reduction in shoot and root growth under oxidative environment is mostly attributed to the excess accumulation of ROS. ROS and RNS are known to be produced in response to environmental stresses [33, 62]. In a study Vanderauwera et al. [63] showed that higher sensitivity to oxidative stress in plants is probably attributed to the high levels of peroxides in addition to other types of ROS. Increased shoot and root growth in *atao3* under nitrosative stress might be attributed to the possible positive role of NO in seed germination and seedling growth, as reviewed extensively by Kopyra and Gwozdz [64]. Similarly, NO has been suggested to break seed dormancy and promote plant growth, even better than GA_3_ [65]. Similarly, by using various NO donors, Giba et al. [66] revealed the possible role of NO in phytochrome-controlled germination of Empress Tree (*Paulownia tomentosa*). This is further supported by increased SNO levels in *atao3* under control and GSNO-mediated nitrosative stress condition while reduced under oxidative stress conditions (Fig. 3e). In contrast, CySNO treated plants showed reduced SNO levels in all genotypes compared to GSNO treated plants, however, compared to WT CySNO treated *atnced3* and *atao3* showed increased SNO accumulation (Fig. 3e). It is somehow strange that both CySNO and GSNO treated plants showed significantly different behavior in accumulation of SNOs, however, this might be due to different NO releasing capacity of both NO donors as suggested by He et al. [67].

As discussed earlier, the promoter analysis of ABA-related genes suggested the presence of *cis*-regulatory elements involved in defense; therefore, we investigated the role of *AtAO3* in basal and *R*-gene-mediated resistance. Our results showed that *AtAO3* negatively regulates both basal and *R*-gene-mediated resistance. The reduced pathogen growth in *atao3* followed by increased *PR1* gene expression (Fig. 4a, b) suggests that *AtAO3* negatively regulates pathogen-induced SA pathway. Transcript accumulation of another SA pathway-related gene *PR2* was reduced compared to that of *PR1*. This might be because of increased *PR1* expression that already contributed to resistance against pathogens and might partly be a strategy to store the extra metabolic energy that would have been used by *PR2* induction. We also observed increased HR and *PR1* expression in response to the avirulent pathogen (*Pst* DC3000 expressing *avrB* effector) in *atao3* plants, indicating that *AtAO3* also negatively regulates *R*-gene-mediated resistance. This strongly supports the hypothesis that *AtAO3* negatively regulates pathogen-induced SA pathway as the SA-deficient mutant *atsid2* showed severe symptom development, increased pathogen growth with reduced *PR1* expression in response to virulent *Pst* DC3000 (Fig. 4a, b, c), and reduced HR and *PR1* expression in response to avirulent *Pst* DC3000 (Fig. 5a, b). Previous studies also suggested resistance of ABA-deficient mutants in response to biotrophic pathogens [45]. Extensive studies have established that plants and their pathogens continuously compete for survival and food. The pathogen-associated molecular patterns (PAMPs) are recognized by plant pattern recognition receptors, leading to PAMP-triggered immunity (PTI) [41]. However, some pathogens have evolved and evade the PTI with modified PAMPs, causing effector-triggered susceptibility. As a part of this strategy, plants have evolved complex defense systems, including plant disease resistance (*R*) genes encoded by the NBS-LRR to recognize pathogen-released effector molecules [41]. Therefore, *R*-gene-mediated resistance is conferred by the interaction between *R*-gene product and *avr* that serves to limit pathogen growth inside the host plants, mostly resulting in HR [68, 69]. Disruption of the SA pathway by mutation (e.g., *NahG* and *sid2*) remarkably compromises resistance toward pathogens [70]. The expression of SAR marker genes suggests that *AtAO3* might have no significant role in SAR. SA, a small phenolic compound, is considered to play a critical role in plant defense. SA has been shown to be required for pathogen recognition, subsequent establishment of local resistance, and finally transmitting signals to the systemic tissues and whole plants [71, 72].

ABA, also known as the “stress hormone,” is a phytohormone, which plays a key role in the regulation of water status and stomatal movement to protect plants under adverse drought conditions [45]. Furthermore, ABA has been shown to positively affect tolerance to particular stresses either by exogenous application or through overexpressing genes that are involved in ABA biosynthesis [73]. Therefore, we intended to determine the role of ABA biosynthetic gene *AtAO3* toward drought tolerance. Our results suggested that both *atao3* and *atnced3* mutants showed rapid wilting, leading to death of plants at 7 days post drought (dpd; Fig. 7a). Similarly, both *atao3* and *atnced3* showed significantly low ABA contents compared to those in WT under drought conditions (Fig. 7c). Earlier wilting in *atao3* and *atnced3* might be due to their inability to close stomata as a part of the adaptation toward the external environment. This was confirmed further when we observed the stomatal structure and found that, in both *atao3* and *atnced3* plants, stomata remained open even after 7 dpd (Fig. 7b) and supported by the quantification of stomatal opening (Additional file 3). This was partially expected as *AtAO3* is involved in ABA biosynthesis, which is mainly considered for the regulation of stomatal movement. Similarly, Christmann et al. [74] reported that ABA is involved in the closure of stomata during drought conditions. This is mainly to limit water loss. This was further supported by our results that the loss of function mutants *atao3* and *atnced3* showed rapid and excessive water loss compared to WT. The results of previous studies also suggest that ABA-controlled processes are significant for the survival of plants, and ABA-deficient mutants are susceptible to water stress [75, 76]. Notably, the ABA contents in *atao3* and WT were almost similar; however, under drought conditions, ABA contents of *atao3* were significantly reduced (Fig. 7c), suggesting that *AtAO3* might be required for ABA biosynthesis only under drought conditions.

We further intended to determine how the loss of function mutation in *AtAO3* affects the expression of other drought stress-related genes. We found that except *ABA3*, which showed no significant difference in transcript accumulation between *atao3* and WT, the remaining ABA biosynthesis-related genes such as *AtABA2* and *AtNCED3* showed reduced transcript accumulation compared to that of WT (Fig. 8 C, G). However, even in the absence of *AtAO3*, some ABA production was noted, indicating that, in the absence of *AtAO3*, other genes such as *AtAO2* can perform this function. Genes that are mostly involved in ABA signaling during drought conditions such as *AtABI2, AtDREB2*, and *AtAPX1* showed induced expression, indicating that these genes respond to drought stress irrespective of the amount of ABA in cells; therefore, they are considered as the key modulators of drought stress [77–79].

Considering that significant crosstalk occurs between redox-active molecules produced in response to biotic and/or abiotic stress conditions, determining the role of NO in modulating these insults would be interesting. NO, because of its physiochemical properties, is a steering molecule that has the potential to regulate the twin (ROS and RNS) redox molecules. However, the mechanistic control by NO seems to be considerably complex as NO can directly control the expression of certain genes and has significant potential to change protein function through post-translational modifications such as *S*- nitrosation [8], tyrosine nitration [80], and methylation [81]. The response of ABA-metabolism-related genes toward NO donor is an indication that NO directly and/or indirectly is involved in drought modulation. However, a thorough investigation is required to elucidate the underlying mechanisms.

## Conclusion

This study suggests a possible role of NO in the regulation of ABA metabolism in plants. Further investigation on the role of ABA biosynthetic gene *AtAO3* showed differential role towards biotic and abiotic stress conditions. *AtAO3* negatively regulates pathogen-induced SA pathway whereas positively regulates drought stress. The biochemical analysis suggested that under normal conditions *AtAO3* may not be required for ABA production however it is required during drought condition as *atao3* plants showed a significant reduction in ABA contents compared to that in WT plants. We also suggested that drought-related genes such as *DREB2* and *ABI2* showed response to drought irrespective of ABA contents.

## Methods

### Transcriptome-wide identification and analysis of ABA metabolism-related genes

Previously, we reported many differentially expressed genes (DEGs) in response to 1 mM CySNO in an RNA-seq-based transcriptomic study Hussain et al. [25]. In this study, we focused on CySNO-induced (both up- and down-regulated until stated otherwise) ABA biosynthesis/metabolism-related genes. The details of plant materials and methodology of RNA extraction and RNA-seq analysis is described in Hussain et al. [25]. Briefly, RNA was extracted from fresh leaves by using RNeasy Plant Mini Kit (Qiagen USA) as per manufacturer’s standard protocol. After RNA integrity and purity were assessed using a Bio-analyzer (Agilent® 2100 Bio-analyzer; Agilent), RNA libraries were generated using TruSeq™ RNA library prep kit (Illumina USA). Next, double-stranded cDNA libraries were synthesized and quantified using KAPA library quantification kit (Illumina USA) and processed for sequencing by using a HiSeq-2500 sequencing machine (Illumina USA). The high-quality reads were identified by processing the raw sequence reads by using a threshold level of Q20 > 40%. The reads with Q20 < 40%, or those with 10% ambiguous bases were discarded [82]. The high-quality reads were then aligned against *A. thaliana* genome by using TopHat with default parameters [83]. The gene annotation was obtained from Ensembl [84], and abundance of the transcripts was confirmed using Cufflinks package v2.2.1 [85]. The expression levels of genes and transcripts under control and treated conditions were calculated using Cuffdiff v.2.2.1 [85] to identify the significant DEGs (Q < 0.05), which were then processed for further analysis.

All the DEGs were mapped against the Arabidopsis database by using MapMan 3.6.0RC as described in [26]. Based on BIN numbers (17.1.1, 17.1.1.1.1, 17.1.2, and 17.1.3) that are specified for ABA metabolism, we identified about 26 DEGs (17 up- and 9 down-regulated) that were involved in ABA metabolism. All the genes were manually checked, and duplicate entries were removed. A heatmap was generated from FPKM values by using *R* version 3.3.1.R (https://www.r-project.org/) to visualize the differences in expression values between treated and control samples, and a dendrogram representing the hierarchical clustering was developed. A multi-dimensional scattering (MDS) plot was also generated from the FPKM values of control and CySNO-treated samples in triplicates to visualize dispersion in the data by using *R*.

### Promoter analysis

ABA-related genes are mostly considered to be involved in abiotic stress only. However, the response of these genes toward NO donors can make them potential targets for biotic stress as well. Therefore, we intended to study the promoter region of CySNO-induced ABA metabolism-related genes for the presence of *cis*-regulatory elements. The promoter sequences of all these genes 1 Kb upstream of the transcription initiation site were retrieved from TAIR (https://www.arabidopsis.org/) and analyzed using PlantCARE (http://bioinformatics.psb.ugent.be/webtools/plantcare/html/). Many *cis*-regulatory elements were found in the promoter region, of which few important ones that were involved in both biotic and abiotic stress conditions were selected; they were then mapped using a pattern matching tool in Regulatory Sequence Analysis Tool [86] package by using default parameters. The resolution of the resultant map was adjusted using Microsoft Power point (https://www.office.com/) and Adobe Photoshop CS6 (https://www.adobe.com/products/photoshop.html). *AtAAO3* (AT2G27150) was selected for the in vivo analysis owing to its key role in ABA biosynthesis pathway. It regulates the last committed step of ABA biosynthesis in which it converts abscisic aldehyde to ABA.

### Plant material and growth conditions

Seeds of Arabidopsis, wild type (WT) Col-0, *atnced3* and *ataao3* (AT2G27150), loss-of-function mutants, were obtained from the Nottingham Arabidopsis Stock Centre (NASC) (http://arabidopsis.info/). The *atgsnor1–3* deficient in ATGSNOR1 was kindly provided by Professor Gary Loake, the University of Edinburgh. Plants were grown either on 1/2 Murashige and Skoog (MS) medium or soil at 23 ± 2 °C, under long-day conditions (16 h light and 8 h dark). Plants were genotyped at the rosette stage (4-week-old plants) to identify homozygous lines. Arabidopsis WT and all other lines used in the study were of the Col-0 genetic background. Changes in the cellular levels of ROS and RNS affect metal-containing enzymes such as peroxidases and catalases [87, 88]. Since we intended to determine the role of *AtAO3* gene under redox stress conditions (oxidative and nitrosative) in plants, we used the *atgsnor1–3* and *atcat2* mutants as a comparative control owing to their established reported role in plant immunity and growth [14, 37]. Similarly, the *atsid2* mutant deficient in SA pathway was used as control for SA-mediated defense pathway [89], and *atnced3* deficient in *AtNCED3* was used for ABA-related defense response [90].

### Redox stress assay

The possible role of *AtAAO3* in redox stress mediation was determined by subjecting WT and KO mutants to both nitrosative and oxidative stress conditions as described earlier [91]. Briefly, the seeds of Arabidopsis WT and *atao3* seeds were surface-sterilized with 50% bleach solution containing 0.1% Triton X-100 (Sigma Aldrich, USA) for 5 min. The seeds were then rinsed with sterile distilled water three times and stratified at 4 °C for 24 h to obtain uniform germination. They were then germinated on 1/2 strength MS medium supplemented with 2 mM H_2_O_2_, 1 μM methyl viologen (oxidative stress), and 1 mM each of GSNO and CySNO (nitrosative stress) in triplicate with at least eight seeds in each replicate. The cotyledon development frequency (CDF, the number of green developed seeds) was recorded after 1 and 2 weeks, and root and shoot length were recorded after 2 weeks of treatment.

### Pathogen growth and inoculation

Two types of bacterial strains, virulent and avirulent *Pseudomonas syringae* pv. *tomato* (*Pst*) were used in this study. Virulent *Pst* DC3000 and avirulent *Pst* DC3000 expressing the *avrB* effector were grown, maintained, and inoculated, as described previously [48]. Briefly, both the bacterial strains were cultured on petri plates containing Luria-Bertani (LB)-agar media with relevant antibiotics for selection (100 mg/mL rifampicin for virulent *Pst* DC3000 and kanamycin (50 mg/mL) and rifampicin for *Pst* DC3000 (avrB). The plates were then incubated at 28 °C overnight, following which a single colony was transferred to LB broth and incubated at 28 °C overnight with continuous shaking. Bacteria were then harvested by centrifugation at 8000 rpm for 3 min in desktop centrifuge (LaboGene Model no 1524 Korea), re-suspended in 10 mM MgCl_2_, and syringe-infiltrated into the abaxial side of leaves at a concentration of 5 × 10^5^ CFU for virulent whereas at 5 × 10^6^ CFU for avirulent strains. Plants were inoculated in triplicates, and three leaves in each plant were inoculated. Control plants were only infiltrated with buffer (10 mM MgCl_2_). Plants were regularly observed for disease symptoms, and leaf samples were collected over time for further analysis.

### Quantitative real-time PCR analysis

The quantitative real-time PCR (qRT-PCR) analysis was performed as described by [61]. Briefly, total RNA was extracted from the inoculated leaves by using Trizol® (Invitrogen, USA) as per manufacturer’s protocol. Complementary DNA (cDNA) was synthesized using the DiaStar™ RT kit (SolGent, Korea) according to manufacturer’s instructions. The synthesized cDNA was then used as a template in a real-time PCR machine (Illumina, USA) to study plant defense marker genes, such as *PR*, which are involved in SA-dependent defense pathway. The detailed list of these genes and their primer sequences are provided in Additional file 4. PCR was performed using the EcoTM real-time PCR machine by using 2× Real-time PCR Master Mix (including SYBR® Green I BioFACT™ Korea) along with 100 ng of templatse DNA and 10 nM of each primer in a final volume of 20 mL. A “no template control” was used as a negative control that contained distilled water instead of template DNA. Two-step PCRs were performed for 40 cycles under the following conditions: polymerase activation at 95 °C for 15 min, denaturation at 95 °C for 15 s, and annealing and extension at 60 °C for 30 s. The melting curves were assessed at 60–95 °C for verification of amplicon specificity for each primer pair, and actin was used as an internal reference gene.

### Histological staining

The hypersensitive response (HR) of WT and *atao3* plants toward *Pst* DC3000 (*avrB*) infiltration was quantified using trypan blue histological staining as described by Koch and Slusarenko [92]. For this, the leaves of WT and *atao3* plants inoculated with *Pst* DC3000 (*avrB*) were stained with lactophenol trypan blue, following which they were de-stained in saturated chloral hydrate. Leaves were then mounted on glass slides in 70% glycerol, and images were captured using a light microscope. Cell death was quantified (arbitrary units) in terms of the intensity of trypan blue staining by using Adobe Photoshop CS6, as described previously [48].

### SNO measurement

Cellular SNO levels after various stresses were assessed as described earlier [61]. Briefly, for plants grown on ½ MS media under oxidative or nitrosative stress (mentioned in detail under subsection “Redox stress assay”), samples were collected 2 weeks after exposure to respective (oxidative and nitrosative) stress conditions. For measuring SNO levels in plants after pathogenicity; 4 weeks old plants were used and samples were collected at 0, 12 and 24 h of attempted *Pst* DC3000 (*avrB*) inoculation. Plant samples (two leaves each pooled as one replicate) were ground in liquid nitrogen and homogenized in PBS buffer. The samples were then centrifuged at 15000 rpm in a refrigerated centrifuge (1736 R LABOGENE Korea; rotor GRF-L-m2.0–30) for 10 min. The supernatant was transferred to a fresh tube and centrifuged again at 15000 rpm for 5 min. About 100 ul of supernatant was injected to the purge vessel of NO analyzer (NOA- 280i, Sievers, and USA) and the peak value was recorded. Total protein contents were determined in all plant samples using the Bradford assay [93]. A standard curve was constructed to measure protein concentration in samples by using Optical density (OD_595_) of bovine serum albumin (BSA) standards. The SNO contents (pmol. μg^− 1^ protein) in samples were calculated by comparing the peak values for each sample with values obtained for the various CySNO standards.

### Drought tolerance assays

About 4-week-old plants were used for drought stress by using the water withholding method [94]. Experimental error was avoided by irrigating the plants with an equal amount of water, and trays were transferred to dried tray bases after complete absorption of water. Plants were subjected to drought for 7 to 10 days. Control plants were watered regularly. The parameters such as electrolyte leakage and stomatal regulation were studied. Transcript accumulation of genes that were potentially involved in drought stress mediation was also studied. The plants were re-watered after 10 days, and the rate of recovery was calculated after 24 h of watering. All the genotypes were assessed for their phenotypic response during drought stress.

### Measurement of electrolyte leakage for cell death quantification

Ion or electrolyte leakage was measured as described by [95] with some modifications. Leaf samples were collected from control and drought stress-treated plants at 0 and 7 days post-drought (dpd) treatment. Leaf discs (1 cm in diameter) were collected in triplicates with at least three leaf discs (from different leaves) in each replicate. The leaf discs were rinsed with deionized water to remove any surface electrolytes and transferred to glass tubes having 5 mL de-ionized water. The tubes were kept at room temperature for 24 h with continuous gentle shaking, and the electrolyte leakage of each sample was measured using a portable conductivity meter (HURIBA Twin Cond B-173; Japan); this was called electrolyte leakage 1 (EL1). The samples were then autoclaved at 120 °C for 20 min and cooled to room temperature (~ 25 °C). The electrolyte leakage of each sample was measured again (EL2). The EL (%) was measured as follows:

EL (%) = (EL1/EL2) × 100.

### Stomatal structure analysis

To determine whether *AtAO3* is involved in stomatal regulation, we investigated the stomatal structure of WT and *atao3* mutants under control and drought conditions, as described previously [96]. Briefly, a thin layer of transparent nail polish was applied on the abaxial surface of the leaves and allowed to dry for a few minutes. Transparent scotch tape was applied carefully on the dried nail polish and pressed gently to have a clear impression of the stomata. The tape was carefully removed from the leaf and mounted on a microscopic slide. The prepared slides were observed under the Axioplan 2 imaging microscope (Axioplan 2 imaging; Carl Zeiss, Jena, Germany). To quantify stomatal opening and to statistically evaluate the stomatal response in WT and transgenic lines after drought stress, we analyzed the images taken with imaging microscope with ImageJ [97]. Briefly, the line tool was selected to measure the scale bar on each image, the scale value was then set to 200 μm. after setting the scale value, the stomata were measured at the widest point from the inside wall of one guard cell to the inside wall of the other. The mean values of eighteen stomata each were calculated and plotted for WT and transgenic lines.

### Measurement of ABA content

The detailed method of Qi et al. [98] was used for the quantification of endogenous ABA. Plants samples collected under control and drought conditions were freeze-dried and grinded to obtain a fine powder, which was then extracted and chromatographed using a standard of ABA (Me-[2H6]-ABA). The fraction of sample was consequently methylated using diazomethane. Finally, ABA was detected and quantified using GCMS (6890 N network gas chromatograph). The signal ions (m/z-162 and m/z-190 for Me-ABA) and (m/z-166 and m/z-194 for Me-[2H6]-ABA) were monitored using ThermoQuset (Manchester, UK).

### Statistical analyses

For all assays the experiments were repeated more than two times and the representative results presented. The data points in redox stress assay were mean of three replicates with five plants pooled in each replicate while for pathogenicity assessment and colony growth the data points were mean of five replicates. For experiments having multiple stresses, the data were subjected to one-way analysis of variance (one-way ANOVA) followed by Duncan’s multiple range test (DMRT) using SAS statistical program version 9.2 (Cary, Nc, United States). The rest of the data were analyzed for standard error (± SE) and student *t*. test to calculate *p* value using Microsoft Excel Program. All the graphs were prepared in Origin 9.0 software.

## Supplementary information

**Additional file 1.** List of ABA metabolism-related genes that showed response to 1 mM CySNO in the RNA-seq-based transcriptome.

**Additional file 2 **Transcript accumulation of *PR2* gene after attempted P*st* DC3000 inoculation.

**Additional file 3.** Quantification of stomatal opening under control and Drought stress condition.

**Additional file 4.** List of genes analyzed using qPCR and their primer sequences.

## Data Availability

All the supporting data can be found as additional file along with this manuscript. Further data analyzed during the current study is available upon reasonable request from corresponding author.

## Abbreviations

ABA: Abscisic acid
AOs: Aldehyde oxidases
AtSABP3: Arabidopsis salicylic acid binding protein 3
AZI: Azelaic acid inducer
BLAST: Basic local alignment search tool
CDF: Cotyledon development frequency
DEGs: Differentially expressed genes
dpd: Days post drought
EL1: Electrolyte leakage 1
G3PDH: Glyceraldehyde 3-phosphate dehydrogenase
GO: Gene ontology
GSH: Glutathione
HR: Hypersensitive response
MS: Murashige and Skoog
NBS-LRR: Nucleotide binding site-leucine rich repeats
NCED: 9-cis-epoxycrotenoid dioxygenase
NPR1: Non-expressor of pathogenesis related gene-1
OH: Hydroxyl radical
PMT: Post-translational modification
qRT-PCR: Quantitative real-time PCR
RNA-Seq: RNA sequencing
RNIs: Reactive nitrogen intermediates
ROIs: Reactive oxygen intermediates
ROS: Reactive oxygen species
SA: Hormone salicylic acid
SAR: Systemic acquired resistance
TAIR: The Arabidopsis Information Resource
TFs: Transcription factors
