# Supplementary material for: Nitric oxide- induced AtAO3 differentially regulates plant defense and drought tolerance in Arabidopsis thaliana
Source: BMC Plant Biol. 2019 Dec 30;19:602. doi: 10.1186/s12870-019-2210-3 (PMC6937950; doi:10.1186/s12870-019-2210-3)
Supplement: Supplementary file 2 — Additional file 2 Transcript accumulation of PR2 gene after attempted Pst DC3000 inoculation. [file 12870_2019_2210_MOESM2_ESM.docx]

**Additional file 2. Transcript accumulation of *PR2* gene after attempted P*st* DC3000 inoculation.**
