# Supplementary material for: Nitric oxide- induced AtAO3 differentially regulates plant defense and drought tolerance in Arabidopsis thaliana
Source: BMC Plant Biol. 2019 Dec 30;19:602. doi: 10.1186/s12870-019-2210-3 (PMC6937950; doi:10.1186/s12870-019-2210-3)
Supplement: Supplementary file 3 — Additional file 3. Quantification of stomatal opening under control and Drought stress condition. [file 12870_2019_2210_MOESM3_ESM.docx]

**Additional file 3. Quantification of stomatal opening under control and Drought stress condition.**

The data points indicate mean stomatal opening of eighteen samples**.** Error bars represents ±SE. Significant differences compared to WT under control and drought (7 days post drought) conditions are represented by asterisks (Student *t*-test). * represents *p* < 0.05, ** represents *p* < 0.01 and *** represents *p* ≤ 0.001.
