# Supplementary material for: Nitric oxide- induced AtAO3 differentially regulates plant defense and drought tolerance in Arabidopsis thaliana
Source: BMC Plant Biol. 2019 Dec 30;19:602. doi: 10.1186/s12870-019-2210-3 (PMC6937950; doi:10.1186/s12870-019-2210-3)
Supplement: Supplementary file 4 — Additional file 4. List of genes analyzed using qPCR and their primer sequences. [file 12870_2019_2210_MOESM4_ESM.docx]

Additional file 4. List of genes analyzed through qPCR and their primer sequences

| S.NO | Name | Forward primer (5´-3´) | Reverse primer (5´-3´) |
| --- | --- | --- | --- |
| 1 | *AtAO3* | GCCAAGACAGTACCAACCTTG | GAGGAGAGACACCAGCAACAG |
| 2 | *AtNCED3* | TCCTCTGTTTCGTTCACGACG | CGTACGGAACCCTTGACGGA |
| 3 | *AtAPX1* | GTCCATTCGGAACAATGAGGTTTGAC | GTGGGCACCAGATAAAGCGACAAT |
| 4 | *AtABI2* | GTTCTTGTTCTGGCGACGGAGC | CCATTAGTGACTCGACCATCAAG |
| 5 | *AtABA2* | ACGGTTGATGATGTAGCGAACGCTGTT | CATCTGAAGACTTTAAAGGAGTGGTTAG |
| 6 | *AtABA3* | CGTCGTCAGTGGAAGGTTTC | AATTTCACCGGTCAGACCCT |
| 7 | *AtDREB1A* | ACGAGTCTTCGGTTCCTCA | ACAAACCCACTTACCGGAGT |
| 8 | *AtDREB2A* | GACCTAAATGGCGACGATGT | TCGAGCTGAAACGGAGGTAT |
| 9 | *PR1* | GTGCAATGGAGTTTGTGGTC | TCACATAATTCCCACGAGGA |
| 10 | *PR2* | CAGATTCCGGTACATCAACG | AGTGGTGGTGTCAGTGGCTA |
| 11 | *AZI* | GCAAGCCAAGTCCTAAACCA | GTCGACGTCAACCAAACCTT |
| 12 | *G3DPH* | CGTCTTTTGGGGAAATCAGA | GACATTGTCAATCGGCACAC |
| 13 | *Actin* | GCTGGACGTGACCTTACTGA | CCATCTCCTGCTCGTAGTCA |
